# Socioeconomic disparities in prevalence, awareness, treatment, and control of hypertension over the life course in China

**DOI:** 10.1186/s12939-017-0597-8

**Published:** 2017-06-13

**Authors:** Fan Yang, Dongfu Qian, Xueyi Liu, for the Healthy Aging and Development Study Group in Nanjing Medical University, for the Data Mining Group of Biomedical Big Data in Nanjing Medical University

**Affiliations:** School of Health Policy & Management, Nanjing Medical University, Hanzhong Road 140, Nanjing, 210029 Jiangsu Province People’s Republic of China

**Keywords:** Hypertension, China, Life Course, Socioeconomic disparities;

## Abstract

**Background:**

The socioeconomically disadvantaged populations are more likely to suffer from hypertension, and few have effectively treated and controlled their hypertension. Research on socioeconomic disparities in prevalence, awareness, treatment, and control of hypertension is warranted to inform the development of new strategies for reducing such health inequities.

**Methods:**

The China Health and Nutrition Survey (CHNS) followed up 20,174 individuals over a 20-year period. We added seven key socioeconomic indicators with age and age-squared into the mixed-effects models to explicitly assess the effect of socioeconomic determinants on hypertension throughout the adult life course.

**Results:**

Prevalence of hypertension was at a higher level in the younger birth cohorts than that in the older generations. Age-related increases in prevalence, awareness, treatment, and control of hypertension were observed over the adult life course. Males, insured and ethnic Han were more likely to suffer from hypertension than their counterparts [coefficient (95% confidence intervals): 0.07(0.04, 0.09), 0.02(0.01, 0.03) and 0.05(0.03, 0.07), respectively]. Hypertension was more prevalent among individuals with higher income who lived in urbanized communities, and less among those with higher education attainment [coefficient (95% confidence intervals): −0.07(−0.12, −0.016)] across adulthood. High-level urbanization and education increased the probabilities of awareness, treatment, and control of hypertension, while household income decreased them [coefficient (95% confidence intervals): 0.28(0.17, 0.39), 0.27(0.17, 0.37) and 0.14(0.08, 0.21), respectively] over the adult life course.

**Conclusions:**

Community urbanicity brought the raise in awareness, treatment, and control of hypertension, but also led to an increase in prevalence of hypertension. People with fewer educational years or higher income may be the disadvantaged population of hypertension over the adult life course in China.

**Electronic supplementary material:**

The online version of this article (doi:10.1186/s12939-017-0597-8) contains supplementary material, which is available to authorized users.

## Background

The prevalence of hypertension in the Chinese adult population increased substantially from around 20 to 34% between 2002 and 2010 [1], and the rates of awareness, treatment, and control of hypertension are still unacceptably low. More seriously, hypertension contributes to health inequalities in China. The socioeconomically disadvantaged populations are more likely to suffer from hypertension [2], and few have effectively treated and controlled their hypertension. Research on socioeconomic disparities in prevalence, awareness, treatment, and control of hypertension is warranted to inform the development of new strategies for reducing such health inequities.

Although previous cross-sectional studies [3–5] have evaluated the health inequities in hypertension burden, they failed to disentangle the effects of adult life course and cohort processes. This is because socioeconomic determinants might vary across age and historical period over the past 30 years of large-scale societal transitions. Thus, the longitudinal data should be used to accurately measure such social disparities in hypertension across time. And to our knowledge, no previous longitudinal study has evaluated the socioeconomic disparities in prevalence, awareness, treatment, and control of hypertension over adult lifespan. Therefore, we research this using data from the China Health and Nutrition Survey (CHNS), which is a large-scale longitudinal survey.

## Related literature

Many cross-sectional studies on socioeconomic disparities in hypertension have been conducted in both developed [6, 7] and developing counties [6, 8–13]. Some studies have also analyzed the possible causes of such socioeconomic disparities [14–17].

A prospective urban–rural epidemiology study [6] conducted data from 153,996 adults from 628 communities in three high-income countries (HIC), ten upper-middle-income and low-middle-income countries (UMIC and LMIC), and four low-income countries (LIC). Results indicated the prevalence of hypertension was higher in men compared to women in HIC, LMIC, and LIC. The rates of hypertension awareness, treatment, and control were higher in urban than that in rural areas of LICs and LMICs, and they were negatively associated with educational achievement in LICs. Kearney PM and colleagues [7] found the variation in rural –urban difference in the level of prevalence, awareness, treatment and control of hypertension across countries. The prevalence of hypertension was higher in urban communities compared to rural ones in India and Thailand. However, hypertension prevalence of rural areas is equal to that of urban areas in Poland, and is even more than that of urban areas in Spain.

From the developing country setting, Luis Rosero-Bixby and WH Dow [11] determined socioeconomic status (SES) gradients in the different dimensions of hypertension and obesity. Results showed obesity and hypertension were worse among high-SES individuals, and lack of exercise and smoking were more common among low SES. Fernald LC and Adler NE [12] conducted a house-to-house cross-sectional survey in low-income regions of rural Mexico, they found household income was positively associated with systolic blood pressure (SBP), but educational achievement was negatively associated with SBP.

In China, the National Nutrition and Health Survey Working Group [8] investigated 141,892 Chinese adults in the 2002. Results indicated the prevalence of hypertension was lower among women than men, but the rates of awareness and treatment were higher among women compared with men. The rates of prevalence, awareness, treatment, and control of hypertension were higher in urban than in rural dwellers. The 2007–2008 China National Diabetes and Metabolic Disorders Study [9] also showed the prevalence of hypertension was higher in urban than in rural areas, and was higher in men than in women. L. Cai and colleagues [13] examined how obesity was distributed across socio-economic gradients in rural China, they found the educational achievement was negatively associated with obesity, and household income was positively associated with central obesity.

Fan Yang [16] conducted an effect evaluation of community intervention trial of chronic disease in China rural areas, and indicated the intervention was more effective in these rural residents with high education than their counterparts. Galdas PM et al. [14] reviewed the key literatures about men’s health-related help seeking behaviour. Results showed many studies highlighted a trend of delay in seeking help among men who become ill, “traditional masculine behaviour” was an explanation for delayed help seeking when they experience illness. Bell CN et al. [15] calculated the association between race/ethnicity and hypertension by levels of social support, and found the race/ethnicity difference of hypertension among those with social support was smaller compared to those without social support; the ethnic difference was only observed among those with social support; Mexican Americans with social support were less likely to suffer from hypertension than their white counterparts. Sebastian Taylor and Alireza Marandi [17] indicated inequities in health services access, use, and benefit are determined by social, political, economic, and cultural factors in China, Cambodia and Iran.

## Methods

The CHNS is a prospective household-based study designed to collect information on key public health risk factors, health outcomes, and demographic, social and economic factors across nine rounds of surveys between 1989 and 2011. Its survey protocols and process for obtaining informed consent were approved by the institutional review committees of the University of North Carolina at Chapel Hill, the National Institute of Nutrition and Food Safety, Chinese Center for Disease Control and Prevention, and the China-Japan Friendship Hospital, Ministry of Health.

The CHNS adopted a multistage, random cluster process [18] to draw the samples samples (Additional file 1: Figure S1). In the first stage, the sampling process was stratified according to geography, economic development, public resources, and health indicators and nine provinces (Heilongjiang, Liaoning, Guangxi, Guizhou, Hubei, Hunan, Henan, Jiangsu, and Shandong provinces) were selected. In the second stage, two cities and four counties were chosen for each province based on income level reported by the National Bureau of Statistics. Except one city was usually the provincial capital, the rest of them were randomly selected [18]. In the third stage, two urban neighbourhoods and two suburban villages were randomly selected within each city; in each selected county, one neighborhood committee was randomly selected from the town where the county government is located, as well as three townships (stratified by income: one high, one low, and two middle-income counties) . One village was randomly selected in each township using the simple random sampling method. The CHNS defined these villages, townships, urban and suburban neighborhoods as ‘communities’, also known as primary sampling units (PSUs). In the final stage, 20 households were selected randomly from each PSU using the household roster and all members in each household were interviewed [18].

The household roster was used to follow-up each of the originally sampled households as well as new households formed from previous households. The follow-up rate was high. The CHNS attempted to follow up with households moving within PSUs or some larger urban entities, rather than the ones from one community to another. Additional details of sampling, response rates, and data quality are reported elsewhere [18–20].

Since only preschoolers and young adults aged 20–45 years were surveyed in 1989, we examined data from CHNS of the 1991, 1993, 1997, 2000, 2004, 2006, 2009, and 2011. Participants were eligible for analysis if they were 18 years or older and their blood pressure was collected, which resulted in an analytic sample of 20,174 individuals (75,729 observations) at multiple exams (mean number of exams: 4).

Data was collected by well-trained health workers at the participants’ houses or a local clinic. Seated systolic/diastolic blood pressure was measured three times by mercury sphygmomanometers on the right arm in triplicate after a 10-min rest according to a standard protocol [21]. The average value of the three measurements was used in our data analysis. Hypertension was defined as an average systolic blood pressure (SBP) of 140 mm Hg or higher and/or an average diastolic blood pressure (DBP) of 90 mm Hg or higher and/or self-reported current use of prescription antihypertensive medications [22]. Awareness of hypertension was defined as self-reported diagnosis of hypertension by a healthcare professional. Treatment of hypertension was defined as self-reported current use of a prescription medication for hypertension management. Control of hypertension was defined as pharmacological treatment of hypertension associated with an average SBP ≤140 mm Hg and an average DBP ≤90 mm Hg. Current drinking was defined as alcohol intake more than once per week during the past 12 months.

The mixed-effects models (models 1, 2, 3) were used to explicitly evaluate the socioeconomic disparities in hypertension because mixed effects models are particularly useful in settings where repeated measurements are made on the same statistical units (longitudinal study) [23]. These models can analysis the relationship between independent variables and a response variable, with coefficients that can change with respect to the grouping variables. We focused on gender, race/ethnicity, marital status, medical insurance, urbanization index, education years and per capita net annual household income over the life course as key socioeconomic indicators. Gender was a dummy variable coded 100 for males and zero for females. Race/ethnicity (100 for ethnic Han, and zero for other minorities), marital status (100 for Married and zero for others), and medical insurance (100 for insured and zero for uninsured) is also represented by the dummy variable. Per capita net annual household income was calculated at the household level for each survey year and inflated to 2011. Urbanization index was calculated at the community level for each survey year by a multicomponent continuous scale [24, 25]. Communities could receive a maximum of ten points for each of 12 components including population density, economic activity, traditional markets, modern markets, transportation infrastructure, sanitation, communications, housing, education, diversity, health infrastructure and social services [24, 25]. We added these key socioeconomic indicators with age and age-squared into the models to identify socioeconomic disparities in prevalence, awareness, treatment, and control of hypertension over the adult life course. The model can be formulated as follows:

1 $$ {Y}_{it} = \left({\beta}_0+{\mu}_{0 i}\right)+\left({\beta}_1+{\mu}_{1 i}\right) A g{e}_{it}+\left({\beta}_2+{\mu}_{2 i}\right) A g{e_{it}}^2+\left({\beta}_3+{\mu}_{3 i}\right) Gende{r}_{it}+\left({\beta}_4+{\mu}_{4 i}\right) Survey\  Y e a{r}_{it}+{e}_{it} $$

2 $$ \begin{array}{l}{Y}_{it} = \left({\beta}_0+{\mu}_{0 i}\right)+\left({\beta}_1+{\mu}_{1 i}\right) Ag{e}_{it}+\left({\beta}_2+{\mu}_{2 i}\right) Ag{e_{it}}^2+\left({\beta}_3+{\mu}_{3 i}\right) Gende{r}_{it}+\\ {}\left({\beta}_4+{\mu}_{4 i}\right) Survey\  Yea{r}_{it}+\left({\beta}_5+{\mu}_{5 i}\right) Gende{r}_{ti}* Ag{e}_{it}+\\ {}\left({\beta}_6+{\mu}_{6 i}\right) Survey\  Yea{r}_{it}* Ag{e}_{it}+{e}_{it}\end{array} $$

3 $$ \begin{array}{l}{Y}_{it} = \left({\beta}_0+{\mu}_{0 i}\right)+\left({\beta}_1+{\mu}_{1 i}\right) Ag{e}_{it}+\left({\beta}_2+{\mu}_{2 i}\right) Ag{e_{it}}^2+\left({\beta}_3+{\mu}_{3 i}\right) Gende{r}_{it}+\\ {}\left({\beta}_4+{\mu}_{4 i}\right) Survey\  Yea{r}_{it}+\left({\beta}_5+{\mu}_{5 i}\right) Gende{r}_{ti}* Ag{e}_{it}+\\ {}\left({\beta}_6+{\mu}_{6 i}\right) Survey\  Yea{r}_{it}* Ag{e}_{it}+\left({\beta}_7+{\mu}_{7 i}\right) Ethnic{y}_{it}+\\ {}\left({\beta}_8+{\mu}_{8 i}\right) Marital\  statu{s}_{it}+\left({\beta}_9+{\mu}_{9 i}\right) Education\  year{s}_{it}+\\ {}\left({\beta}_{10}+{\mu}_{10 i}\right) Urbanicit{y}_{it}+\left({\beta}_{11}+{\mu}_{11 i}\right) Household\  Incom{e}_{it}+\\ {}\left({\beta}_{12}+{\mu}_{12 i}\right) Urbanicit{y}_{it}* Household\  Incom{e}_{it}+\\ {}\left({\beta}_{13}+{\mu}_{13 i}\right) Medical\  insurenc{e}_{it}+\left({\beta}_{14}+{\mu}_{14 i}\right) Current\  drinkin{g}_{it}+{e}_{it}\end{array} $$

where *Y*_*it*_ is the response vectoxr for individual i at time t (the measurement instance for hypertension); *μ*_0*i*_, *μ*_1*i*_ ⋯, *μ*_*ki*_ are the differences between *β*_0_, *β*_1_ ⋯, *β*_k_ and the intercept or slopes of subject *i* (random effects); *β*_0_, *β*_1_ ⋯, *β*_k_are the subjects mean intercept or slopes for the above explanatory variables (fixed effects); *e*_*it*_ is the random error within subjects throughout the adult life course.

The coefficients in the Equation (1), Equation (2) and Equation (3) can be interpreted as age effect, period effect, and the influence of socioeconomic disparities in prevalence, awareness, treatment, and control of hypertension over the life course, respectively. Trajectories of the probability of hypertension prevalence across the adult life course for 1582 participants with measurements for all eight surveys among men and women was estimated by unadjusted mixed effects models stratified by baseline age group (cohort). All statistical tests were 2-sided and were performed using Stata version 12.

## Results

**Fig. 1:**
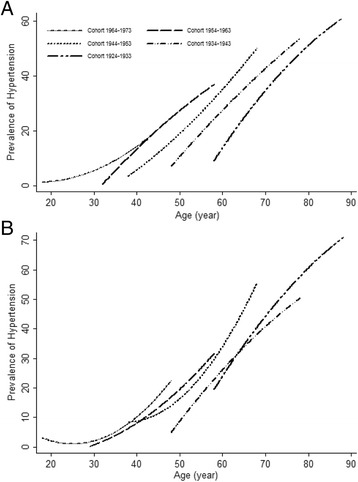
Trajectories of the probability of hypertension prevalence (%) across the life course for 1582 participants with measurements for all 8 surveys among men (A) and women (B) adult, estimated by multilevel mixed effects models stratified by baseline age group (cohort)

The sample characteristics were shown in Table 1. Curvilinear age effects were observed in prevalence, awareness, treatment, and control of hypertension (All *P* <0.0001) over the adult life course (Table 2, Model 1 and see Additional file 2: Table S1). Results of the complete-case analysis confirmed that prevalence of hypertension increased non-linearly with age at any given cohort (Fig. 1). Unadjusted linear mixed effects model stratified by age and gender shows that prevalence of hypertension was higher in younger birth cohorts than in older ones, particularly for men. For example, the prevalence of hypertension among males was higher in the 1934–1943 cohort than that in the 1924–1933 cohort when they were 60–80 years old, but lower than that in 1944–1953 cohort when 50–70 years old.

**Table 1:** Descriptive characteristics of adult population (age > =18) who participated in the 1991–2011 China Health and Nutrition Survey ^a^

	Survey Year							
	1991	1993	1997	2000	2004	2006	2009	2011
Participated	8667	7133	5290	6442	6935	7182	6716	7500
New participated		1096	3368	3071	2335	2008	2852	5134
With drowal/Died		1534	2939	2216	2578	2088	2474	2068
Age	40.1(16.4)	40.6(16.5)	41.8(16.7)	42.5(16.6)	44.3(17.0)	43.5(16.8)	44.5(16.9)	46.0(16.6)
Gender	48.6	48.8	49.0	48.9	48.9	47.3	46.7	47.0
Ethnicy	94.3	94.3	94.7	94.9	94.9	94.8	94.9	96.1
Marital status	75.5	74.8	74.6	75.1	82.1	83.5	83.3	84.1
Education year	15.6(9.7)	16.2(9.5)	17.0(9.3)	18.1(9.1)	18.8(8.8)	18.8(9.4)	19.1(9.0)	20.6(8.9)
Medical insurance	31.3	25.7	25.5	21.7	26.8	49.3	90.7	94.8
Household income ^c^	3036(2264)	3457(2970)	4257(3561)	5556(5686)	7149(7605)	8112(11821)	11579(15164)	13887(16374)
Community urbanicity ^d^	46.1(16.2)	47.6(16.4)	52.3(18.0)	58.2(18.1)	61.4(20.2)	62.6(20.0)	65.5(19.0)	69.5(19.4)
Current drinking	22.4	24.2	25.1	25.2	23.2	23.0	20.9	21.2
Prevalence	14.4	15.8	20.0	21.0	24.0	23.3	29.4	27.9
Awareness	27.4	27.6	21.3	32.6	36.4	42.5	42.8	55.3
Treatment	16.0	16.4	14.4	22.9	27.7	33.1	35.1	47.6
Control	3.0	3.1	2.8	5.3	8.0	8.8	9.2	17.6

^a^Values presented as numbers for arbitrary values and as mean ± SD or % for other variables

^c^Net annual. Inflated to 2011

^d^Measured at the community level on a 12-component continuous scale ranging from 0–120 with higher values corresponding to higher levels of urbanicity

**Table 2:** Coefficients (95% confidence intervals) from mixed effects models (model 1, 2 and 3) predicting of the probability of Hypertension Prevalence over the Life Course among China adults

	Model 1	Model 2	Model 3
Age	0.19(0.08,0.29)	0.26(0.15,0.38)	0.27(0.13,0.42)
Age^2^	0.008(0.007,0.009)	0.007(0.006,0.008)	0.006(0.005,0.008)
Gender	0.05(0.04,0.05)	0.08(0.06,0.10)	0.07(0.04,0.09)
1993	0.78(−0.22,1.78)	2.06(−0.81,4.94)	0.78(−2.39,3.95)
1997	3.62(2.60,4.63)	−0.80(−3.79,2.19)	−1.42(−4.79,1.94)
2000	2.98(1.98,3.98)	−2.16(−5.19,0.88)	−3.36(−6.94,0.21)
2004	3.15(2.12,4.17)	−2.25(−5.50,0.99)	−3.67(−7.20,-0.13)
2006	1.23(0.19,2.27)	−0.63(−3.97,2.72)	−2.75(−6.40,0.91)
2009	6.51(5.46,7.55)	−1.32(−4.71,2.06)	−4.47(−8.91,-0.75)
2011	3.98(2.97,4.98)	−3.21(−6.48,0.05)	−7.33(−10.96,-3.70)
Gender*Age		−0.07(−0.12,-0.03)	−0.05(−0.10,-0.004)
1993*Age		−0.03(−0.09,0.04)	0.01(−0.06,0.08)
1997*Age		0.11(0.04,0.17)	0.12(0.05,0.19)
2000*Age		0.12(0.05,0.19)	0.14(0.06,0.22)
2004*Age		0.12(0.06,0.19)	0.16(0.08,0.23)
2006*Age		0.05(−0.02,0.12)	0.09(0.01,0.16)
2009*Age		0.17(0.10,0.24)	0.20(0.12,0.28)
2011*Age		0.16(0.09,0.22)	0.20(0.13,0.28)
Community urbanicity			0.07(0.05,0.10)
Ethnicy			0.05(0.03,0.07)
Marital status			−0.02(−0.03,-0.01)
Education years			−0.07(−0.12,-0.016)
Medical Insurance			0.02(0.01,0.03)
Household income			1.68E^−4^ (6.21E^−5^, 2.75E^−4^)
Urbanicity*household income			−1.89E^−6^ (−3.26E^−6^, −5.17E^−7^)
Current Drinking			0.010(0.002,0.018)

Hypertension was more prevalent among the males, insured and ethnic Han than their counterparts across adulthood [coefficient (95% confidence intervals): 0.07(0.04, 0.09), 0.02(0.01, 0.03) and 0.05(0.03, 0.07), respectively; all *P* <0.0001] (Table 2, Model 3). Hypertension prevalence increased with community urbanicity and household income (*P* < 0.0001 and *P* = 0.002, respectively), but decreased with education years [coefficient (95% confidence intervals): −0.07(−0.12, −0.016); *p* = 0.009] throughout the adult life course (Table 2, Model 3). Awareness, treatment, and control of hypertension were higher in ethnic Han than in ethnic minorities (*P* = 0.001, *P* = 0.001 and *P* = 0.027, respectively) (Table 3). Awareness, treatment, and control of hypertension increased with urbanization (all *P* <0.0001). Awareness, treatment, and control of hypertension increased decreased with household income (*P* = 0.043, *P* = 0.013 and *P* < 0.0001, respectively), but increased with education years [coefficient (95% confidence intervals): 0.28(0.17, 0.39), 0.27(0.17, 0.37) and 0.14(0.08, 0.21), respectively; all *P* <0.0001)] over adulthood.

**Table 3:** Coefficients (95% confidence intervals) from mixed effects models (model 3) predicting of the probability of Awareness, Treatment, and Control of Hypertension over the Life Course among China adults

	Awareness	Treatment	Control
Age	1.93(1.51,2.36)	1.71(1.31,2.11)	0.62(0.35,0.88)
Age^2^	−0.016(−0.019,-0.012)	−0.013(−0.016,-0.009)	−0.006(−0.008,-0.003)
Gender	−0.05(−0.12,0.03)	0.002(−0.07,0.07)	−0.04(−0.08,0.01)
1993	−13.73(−26.83,-0.62)	−4.54(−16.96,7.88)	0.52(−8.27,9.31)
1997	−19.74(−32.63,-6.86)	−4.39(−16.61,7.82)	−3.64(−12.18,4.89)
2000	−23.66(−36.90,-10.41)	−14.99(−27.53,-2.45)	−8.21(−16.99,0.57)
2004	−31.02(−43.81,-18.23)	−21.22(−33.34,-9.09)	−8.45(−16.86,-0.03)
2006	−31.10(−44.24,-17.96)	−21.56(−33.99,-9.12)	−7.68(−16.30,0.93)
2009	−31.08(−43.89,-18.27)	−25.83(−37.95,-13.71)	−7.70(−16.02,0.63)
2011	−24.71(−37.53,-11.89)	−20.53(−32.69,-8.37)	−13.75(−22.06,-5.45)
Gender*Age	−0.03(−0.16,0.09)	−0.10(−0.21,0.02)	0.02(−0.05,0.10)
1993*Age	0.28(0.05,0.51)	0.10(−0.12,0.32)	−0.02(−0.17,0.13)
1997*Age	0.24(0.01,0.46)	0.03(−0.19,0.24)	0.04(−0.11,0.19)
2000*Age	0.44(0.21,0.67)	0.30(0.08,0.52)	0.15(−0.01,0.30)
2004*Age	0.60(0.38,0.82)	0.44(0.24,0.65)	0.16(0.02,0.31)
2006*Age	0.68(0.45,0.90)	0.52(0.31,0.73)	0.16(0.01,0.31)
2009*Age	0.71(0.49,0.93)	0.64(0.43,0.84)	0.16(0.01,0.30)
2011*Age	0.74(0.52,0.96)	0.68(0.47,0.88)	0.36(0.22,0.50)
Community urbanicity	0.34(0.29,0.40)	0.36(0.31,0.41)	0.12(0.08,0.15)
Ethnicy	0.08(0.03,0.13)	0.07(0.03,0.12)	0.03(0.004,0.06)
Marital status	−0.01(−0.04,0.01)	−0.02(−0.04,0.004)	−0.003(−0.02,0.01)
Education years	0.28(0.17,0.39)	0.27(0.17,0.37)	0.14(0.08,0.21)
Medical Insurance	−0.004(−0.02,0.02)	0.001(−0.02,0.02)	−0.002(−0.01,0.01)
Household income	−2.5 E^−4^ (−4.9E^−4^, −8.24E^−6^)	−2.9 E^−4^ (−5.1E^−4^, −6.1E^−5^)	−4.3 E^−4^ (−5.9E^−4^, −2.8E^−4^)
Urbanicity*household income	5.14 E^−6^ (2.07E^−6^, 8.22E^−6^)	6.12 E^−6^ (3.21E^−6^, 9.04E^−6^)	7.72(5.71E^−6^, 9.72E^−6^)E^−6^
Current Drinking	−0.033(−0.052,-0.014)	−0.051(−0.069,-0.033)	−0.015(−0.027,-0.003)

## Discussion

In this study, we evaluated the socioeconomic disparities in prevalence, awareness, treatment, and control of hypertension over the adult life course with CHNS data. To our knowledge, few studies [8, 9] have definitively disentangled the effect of life course. The present study provides the first longitudinal analyses to document socioeconomic disparities in the burden of hypertension across the adult life course. The findings revealed age-related increases in prevalence, awareness, treatment, and control of hypertension over the adult life course. Younger generations were more likely to suffer from hypertension than the older birth cohorts. Furthermore, women, ethnic minority, and population with high educational level or low household income had a lower probability of suffering from hypertension. Awareness, treatment, and control of hypertension increased with educational level, but decreased with household income throughout the adult life course. Prevalence, awareness, treatment, and control of hypertension increased with community urbanicity over the adult life course.

Our longitudinal analyses found prevalence of hypertension was higher in younger than the older birth cohorts. This may be due to lack of physical activity among younger generations, which leads to higher probability of being obese [23, 26, 27]. Hypertension prevalence was greater for men than women over adulthood, but females were not more likely to be aware of, treat and control their hypertension than males over the adult life time in our study, which is not consistent with other studies [6, 8, 9, 14]. The reason of the different results may be that our longitudinal analysis controlled for age, period and socioeconomic determinants. It implied that socioeconomic factors possibly result in the low rate of hypertension awareness, treat and control among women.

Many studies showed race/ethnic differences in hypertension [8]. We found China ethnic minorities had lower prevalence of hypertension than ethnic Han, which may be attributable to the distributional difference of risk factors between ethnic Han and minorities, e.g. obesity [28]. However, because China ethnic minorities live in the less affluent areas with limited health care resources, they were less likely to have their hypertension diagnosed, treated and controlled. Other studies also indicated that lack of social support may cause the race/ethnicity differences of hypertension [15]. Although medical insurance can improve access to health care, we did not find significant differences between the insured and uninsured in the awareness, treatment and control of hypertension.

Community urbanicity brought the raise in awareness, treatment, and control of hypertension, but also led to an increase in prevalence of hypertension, which are consistent with other China cross sectional surveys [8]. The 2002 China National Nutrition and Health Survey also showed the urban–rural disparity in hypertension. However, hypertension burden of rural areas was equal to or even more than that of urban areas in the developed countries [10]. The reason for the mixed results may be that obesity and less physical activity are more in the urban population compared with rural population in China [29]. On the other hand, urbanization can increase the health resources to bring the raise in awareness, treatment, and control of hypertension in urban areas. Conversely, most hypertensive patients have not been diagnosed and there was a great proportion of lost to follow-up for diagnosed hypertension in rural areas.

Our study showed prevalence of hypertension decreased with education years, and hypertension with more education years also had higher rates of hypertension awareness, treatment and control, which was consistent with evidences from other developing countries [6, 12]. People with high education level tend to have fewer risk factors, and were more likely to change their unhealthy behaviors due to better access to health knowledge [16]. However, our research showed hypertension was more prevalent in high-income population. In other developing areas, low income was also found to be associated with lower SBP in the older Costa Rican [11] and in rural Mexico [12]. A Chinese study has also showed obesity increased with yearly household income [13]. Awareness, treatment and control of hypertension decreased with household income in our study, although some studies showed the income could increase the accessibility and utilization of health care services [17]. Our result contradicts several western studies because of the different stages of social development between developing and the developed countries. In China, rich people are more likely to choose unhealthy lifestyle, and thus more likely to be obese.

There are some limitations in our study. Firstly, the main limitation includes the loss-to-follow-up data. Secondly, some risk factors were unavailable, including family history of hypertension and smoking.

## Conclusion

We observed age-related increases in prevalence, awareness, treatment, and control of hypertension over the adult life course; and prevalence of hypertension was at higher levels in the younger birth cohorts than in the older generations. Community urbanicity brought the raise in awareness, treatment, and control of hypertension, but also led to an increase in prevalence of hypertension. People with fewer educational years or higher income may be the disadvantaged population of hypertension in China. Hypertension prevention should address these socioeconomic determinants to reduce such health inequities.

## Additional files

Additional file 1: Figure S1. The CHNS multi-stage cluster random sampling scheme. (DOCX 175 kb)

Additional file 2: Table S1. Coefficients (95% confidence intervals) from mixed effects models (model 1 and 2) predicting of the probability of Awareness, Treatment, and Control of Hypertension over the Life Course among China adults. (DOC 56 kb)

## Abbreviations

CHNS: China Health and Nutrition Survey
DBP: Diastolic blood pressure
SBP: Systolic blood pressure
