# Supplementary material for: Socioeconomic disparities in prevalence, awareness, treatment, and control of hypertension over the life course in China
Source: Int J Equity Health. 2017 Jun 13;16:100. doi: 10.1186/s12939-017-0597-8 (PMC5470255; doi:10.1186/s12939-017-0597-8)
Supplement: Supplementary file 1 — The CHNS multi-stage cluster random sampling scheme. (DOCX 175 kb) [file 12939_2017_597_MOESM1_ESM.docx]

Fig S1 The CHNS multi-stage cluster random sampling scheme
