# Supplementary material for: Socioeconomic disparities in prevalence, awareness, treatment, and control of hypertension over the life course in China
Source: Int J Equity Health. 2017 Jun 13;16:100. doi: 10.1186/s12939-017-0597-8 (PMC5470255; doi:10.1186/s12939-017-0597-8)
Supplement: Supplementary file 2 — Coefficients (95% confidence intervals) from mixed effects models (model 1 and 2) predicting of the probability of Awareness, Treatment, and Control of Hypertension over the Life Course among China adults. (DOC 56 kb) [file 12939_2017_597_MOESM2_ESM.doc]

**Table S1**. Coefficients (95% confidence intervals) from mixed effects models (model 1 and 2) predicting of the probability of Awareness, Treatment, and Control of Hypertension over the Life Course among China adults.

|  | **Awareness** | | |  | **Treatment** | | |  | **Control** | | |
| --- | --- | --- | --- | --- | --- | --- | --- | --- | --- | --- | --- |
|  | **Model 1** |  | **Model 2** |  | **Model 1** |  | **Model 2** |  | **Model 1** |  | **Model 2** |
| Age | 1.86(1.54,2.19) |  | 1.89(1.51,2.28) |  | 1.34(1.03,1.65) |  | 1.61(1.25,1.97) |  | 0.56(0.36,0.76) |  | 0.54(0.31,0.77) |
| Age^2^ | -0.011(-0.014,-0.008) |  | -0.016(-0.019,-0.012) |  | -0.006(-0.009,-0.004) |  | -0.012(-0.015,-0.009) |  | -0.004(-0.006,-0.002) |  | -0.005(-0.007,-0.003) |
| Gender | -0.06(-0.08,-0.04) |  | -0.07(-0.14,-0.001) |  | -0.06(-0.07,-0.04) |  | -0.02(-0.09,0.04) |  | -0.02(-0.03,-0.01) |  | -0.05(-0.09,-0.01) |
| 1993 | 2.32(-0.82,5.47) |  | -9.31(-21.48,2.87) |  | 1.76(-1.20,4.72) |  | -1.77(-13.27,9.72) |  | 0.04(-2.02,2.10) |  | -0.05(-7.92,7.81) |
| 1997 | -3.85(-6.87,-0.82) |  | -16.80(-28.70,-4.91) |  | -0.07(-2.97,2.78) |  | -3.21(-14.45,8.04) |  | -0.35(-2.31,1.60) |  | -2.76(10.38,4.85) |
| 2000 | 6.00(3.07,8.94) |  | -16.05(-27.93,-4.16) |  | 7.21(4.44,9.98) |  | -8.46(-19.71,2.79) |  | 2.02(0.12,3.92) |  | -5.21(-12.82,2.40) |
| 2004 | 9.08(6.17,11.98) |  | -22.50(-34.57,-10.44) |  | 11.37(8.63,14.11) |  | -13.23(-24.64,-1.82) |  | 4.43(2.56,6.30) |  | -5.70(-13.38,1.98) |
| 2006 | 14.66(11.72,17.59) |  | -25.35(-37.76,-12.94) |  | 16.52(13.76,19.29) |  | -15.47(-27.19,-3.76) |  | 5.19(3.30,7.70) |  | -5.64(-13.52,2.23) |
| 2009 | 17.42(14.59,20.26) |  | -22.61(-34.59,-10.64) |  | 20.70(18.02,23.37) |  | -16.71(-28.01,-5.41) |  | 5.70(3.89,7.51) |  | -5.25(-12.78,2.28) |
| 2011 | 28.77(25.99,31.55) |  | -13.60(-25.60,-1.59) |  | 31.83(29.20,34.45) |  | -8.99(-20.36,2.39) |  | 13.61(11.85,15.38) |  | -7.99(-15.52,-0.46) |
| Gender*Age |  |  | 0.02(-0.09,0.13) |  |  |  | -0.05(-0.16,0.05) |  |  |  | 0.06(-0.01,0.12) |
| 1993*Age |  |  | 0.21(-0.01,0.42) |  |  |  | 0.06(-0.13,0.26) |  |  |  | 0.001(-0.14,0.14) |
| 1997*Age |  |  | 0.24(0.03,0.44) |  |  |  | 0.06(-0.13,0.26) |  |  |  | 0.04(-0.09,0.18) |
| 2000*Age |  |  | 0.40(0.20,0.61) |  |  |  | 0.29(0.10,0.48) |  |  |  | 0.13(-0.003,0.26) |
| 2004*Age |  |  | 0.57(0.36,0.78) |  |  |  | 0.45(0.25,0.64) |  |  |  | 0.18(0.05,0.31) |
| 2006*Age |  |  | 0.71(0.50,0.92) |  |  |  | 0.57(0.37,0.77) |  |  |  | 0.19(0.06,0.33) |
| 2009*Age |  |  | 0.71(0.51.0.91) |  |  |  | 0.66(0.47,0.85) |  |  |  | 0.20(0.07,0.32) |
| 2011*Age |  |  | 0.74(0.54,0.95) |  |  |  | 0.71(0.52,0.90) |  |  |  | 0.37(0.24,0.50) |
